# Boosting the acetol production in methanotrophic biocatalyst *Methylomonas* sp. DH-1 by the coupling activity of heteroexpressed novel protein PmoD with endogenous particulate methane monooxygenase

**DOI:** 10.1186/s13068-022-02105-1

**Published:** 2022-01-17

**Authors:** Tin Hoang Trung Chau, Anh Duc Nguyen, Eun Yeol Lee

**Affiliations:** Department of Chemical Engineering (BK21 FOUR Integrated Engineering Program), Kyung Hee University, 17104, Yongin-si, Gyeonggi-do South Korea

**Keywords:** Acetol production, Acetone oxidation, Coupling activity, *Methylomonas* sp. DH-1, *Methylacidiphilum* sp. IT6, PmoD, pMMO, Whole-cell biocatalyst

## Abstract

**Background:**

*Methylacidiphilum* sp. IT6 has been validated its C3 substrate assimilation pathway via acetol as a key intermediate using the PmoCAB3, a homolog of the particulate methane monooxygenase (pMMO). From the transcriptomic data, the contribution of PmoD of strain IT6 in acetone oxidation was questioned. *Methylomonas* sp. DH-1, a type I methanotroph containing *pmo* operon without the existence of its *pmoD*, has been deployed as a biocatalyst for the gas-to-liquid bioconversion of methane and propane to methanol and acetone. Thus, *Methylomonas* sp. DH-1 is a suitable host for investigation. The PmoD-expressed *Methylomonas* sp. DH-1 can also be deployed for acetol production, a well-known intermediate for various industrial applications. Microbial production of acetol is a sustainable approach attracted attention so far.

**Results:**

In this study, bioinformatics analyses elucidated that novel protein PmoD is a C-terminal transmembrane–helix membrane with the proposed function as a transport protein. Furthermore, the whole-cell biocatalyst was constructed in *Methylomonas* sp. DH-1 by co-expression the PmoD of *Methylacidiphilum* sp. IT6 with the endogenous pMMO to enable acetone oxidation. Under optimal conditions, the maximum accumulation, and specific productivity of acetol were 18.291 mM (1.35 g/L) and 0.317 mmol/g cell/h, respectively. The results showed the first coupling activity of pMMO with a heterologous protein PmoD, validated the involvement of PmoD in acetone oxidation, and demonstrated an unprecedented production of acetol from acetone in type I methanotrophic biocatalyst. From the data achieved in batch cultivation conditions, an assimilation pathway of acetone via acetol as the key intermediate was also proposed.

**Conclusion:**

Using bioinformatics tools, the protein PmoD has been elucidated as the membrane protein with the proposed function as a transport protein. Furthermore, results from the assays of PmoD-heteroexpressed *Methylomonas* sp. DH-1 as a whole-cell biocatalyst validated the coupling activity of PmoD with pMMO to convert acetone to acetol, which also unlocks the potential of this recombinant biocatalyst for acetol production. The proposed acetone-assimilated pathway in the recombinant *Methylomonas* sp. DH-1, once validated, can extend the metabolic flexibility of *Methylomonas* sp. DH-1.

**Supplementary Information:**

The online version contains supplementary material available at 10.1186/s13068-022-02105-1.

## Background

Acetol (hydroxyacetone), with hydroxy and carbonyl groups, is an important intermediate for the production of various industrial materials from the textile to cosmetic industries [1, 2]. The chemical synthesis of acetol is mostly from glycerol dehydration, less commonly from dehydrogenation of propylene glycol or sugar alcohols [1]. However, chemically synthesis processes increase the production price of acetol and constraint its applications [1, 3]. Biological production of acetol, a greener production approach without extreme conditions and toxic solvents, has drawn attention. However, to date, limited studies on the bioconversion of acetol have been published. Patent issued by METabolix EXplorer claimed that the production of acetol from glucose in their engineered *Escherichia coli* reached the titer of 1.63 g/L [2]. Acetol is also produced from glycerol by deploying the methylglyoxal bypass pathway, in which glycerol is converted to dihydroxyacetone phosphate, then to methylglyoxal, and finally to acetol and 1,2-propanediol [3]. *E. coli* Lin43 strain has been metabolically engineered to achieve the acetol titer of 1.82 g/L [4]. Various approaches were deployed to enhance the titer up to 2.81 g/L [5]. Apart from glycerol and glucose, acetol can be converted from acetone by acetone monooxygenase [6]. Acetone monooxygenases were first discovered in actinomycete and mycobacteria. The well-known one is acetone monooxygenase in the propane-utilizing actinomycete *Gordinia* sp. strain TY-5, which belongs to the Baeyer–Villiger monooxygenase family [7]. Another kind of acetone monooxygenase is the four-subunit MimABCD diiron monooxygenase discovered in mycobacteria, *Mycobacterium smegmatis* strain mc^2^155, and *Mycobacterium goodii* strain 12,523 [6, 8]. This acetone monooxygenase can convert acetone to acetol for further assimilation in these mycobacteria with the first biochemical evidence obtained using recombinant *E. coli* whole-cell assays. Besides, the acetone oxidation to acetol can be catalyzed by cytochrome P450. Acetone oxidation catalyzed by cytochrome P450 has been only demonstrated in mammals [9, 10] and recently in type II versatile methanotroph *Methylocella silvestris* [11]. The oxidation of acetone to acetol by acetone monooxygenase and cytochrome P450 is the one-step bioconversion which is simpler than the methylglyoxal bypass pathway. Moreover, the deployment of acetone, a by-product in the acetone–butanol–ethanol (ABE) fermentation process, as the substrate for the bioconversion of acetol enhances the economic values of acetone and widens the possibilities for acetol production.

Recently, *Methylacidiphilum* sp. IT6, a newly isolated thermoacidophilic aerobic methane oxidizer of the phylum Verrucomicrobia, was demonstrated to contain a 2-propanol assimilation pathway which allows its assimilation on C3 substrates as propane oxidation intermediates (2-propanol, acetone, andacetol) [12]. Further analyses validated the role of a proposed acetone monooxygenase (PmoCAB3), a homolog of particulate methane monooxygenase (pMMO), with strict substrate specificity. When *Methylacidiphilum* sp. IT6 was cultivated on different C3 substrates, similar expression profiles were recorded but different from those cultivated on methane in which the *pmoCAB3* showed a high expression under C3 substrate-cultured conditions, which is from 14- to 40-fold higher than that in methane-cultured conditions. The acetone-induced resting cells also demonstrated the specific oxidation to ketones rather than alkanes. These results provided clear evidence to elucidate PmoCAB3 as acetone monooxygenase. Among remaining genes in the C3 substrate assimilation cluster, the hypothetical gene *pmoD* next to the *pmoCAB3*, under acetone-cultured conditions, has a significantly high expression with a 12–24-fold higher than that in methane-cultured conditions. Furthermore, under oxygen-depleted conditions, the volcano plot showed that hypothetical protein PmoD has the highest difference in gene expression between acetone- and methane-cultured cells, much higher than those of other genes in the C3 substrate-assimilation cluster [12]. These results raised a hypothesis of this novel protein PmoD may contribute to acetone oxidation of PmoCAB3. Still, further engineering techniques, such as partial deletions and heteroexpression, should be done to further clarify the specific functions.

Although many strains of the genus *Methylacidiphilum* have been isolated and studied for their characteristics and potentials for more than a decade [13], there is no report to date about the development of genetic tools to metabolically engineer these strains. Thus, deployment of other host strains for further studies of novel monooxygenase and related enzymes of *Methylacidiphilum* sp. IT6 is considered.

Despite being one of the most common host strains for protein expression, *Escherichia coli* has been failed to express pMMO of methanotrophs due to its toxicity to *E. coli* [14, 15]. Thus, to clarify the role of PmoD in acetone oxidation, *Methylomonas* sp. DH-1, a type I methanotroph containing *pmo* operon without the existence of its *pmoD*, is considered a suitable host for investigation. Owing to the fast growth rate, highly efficient conversion [16, 17], and the availability of its genome sequence, genetic tools, and omics data [18, 19], *Methylomonas* sp. DH-1 demonstrated its advantages as a platform strain for the production of various value-added products from methane [20, 21]. Furthermore, *Methylomonas* sp. DH-1, owing to the biocatalytic capacity of its pMMO, has been deployed as a whole-cell biocatalyst for the gas-to-liquid bioconversion of methane to methanol, and propane to 2-propanol or further oxidation by alcohol dehydrogenase into acetone [16, 17, 22]. To date, there is no report on acetone assimilation in *Methylomonas* sp. DH-1. Therefore, the proteobacterial methanotroph *Methylomonas* sp. DH-1 was deployed to validate our hypothesis on the involvement of PmoD in pMMO activity to oxidize acetone into acetol, which, once validated, can be also further developed as a whole-cell biocatalyst for the production of acetol.

In this study, using various bioinformatic tools, the novel protein PmoD of *Methylacidiphilum* sp. IT6 was elucidated as a membrane protein. We also constructed a novel biocatalyst by integrating the *pmoD* to the *pmo* operon of *Methylomonas* sp. DH-1 to validate the involvement of PmoD in enabling the acetone oxidation activity of pMMO, from which PmoD was proposed to function as a transport protein to deliver acetone toward pMMO for oxidation. Besides, under batch conditions, fascinating results of the acetone oxidation shed a light on the possible existence of the C3 substrate assimilation pathway in *Methylomonas* sp. DH-1 when unlocking the bottleneck step, acetone oxidation. Our study provided auxiliary information to further validate the catalytic activity of the proposed acetone monooxygenase PmoCAB3 in strain IT6, developed a novel whole-cell methanotrophic biocatalyst for bioproduction of acetol, and introduced fascinating hypotheses worth further study.

## Results

### Bioinformatics analyses of the protein PmoD from *Methylacidiphilum* sp. IT6

The protein PmoD (GenBank accession number QSR88567) is a hypothetical protein that locates next to the PmoCAB3 cluster in *Methylacidiphilum* sp. IT6, with no information about its structure or functions. Thus, at first, we deployed various bioinformatics tools to perform preliminary elucidations of the novel protein PmoD, and the subunits of PmoCAB3 were also analyzed.

The three subunits, PmoC3, PmoA3, and PmoB3, were aligned with the corresponding components of one representative strain of the *Methylacidiphilum* genus, *Methylacidiphilum kamchatkense* Kam1, one representative strain of the proteobacterial methanotroph, *Methylotuvimicrobium alcaliphilum* 20Z, and this study’s host strain, *Methylomonas* sp. DH-1. Furthermore, their percent identity was also evaluated using BLASTP (Additional file 1: Fig. S1). The BLASTP results of each subunit in the operon showed a high percent identity in their sequences. Among the three reference strains, all subunits of *Methylacidiphilum* sp. IT6 showed a high percent identity with those from *M. kamchatkense* Kam1 with a percent identity above 85%. For the two remaining strains, the values are more divergent with the percent identity fluctuating around 30–40%. The results are consistent with the previous study, in which this PmoCAB3 in strain IT6 was claimed as a homolog of particulate methane monooxygenase [12].

**Fig. 1:**
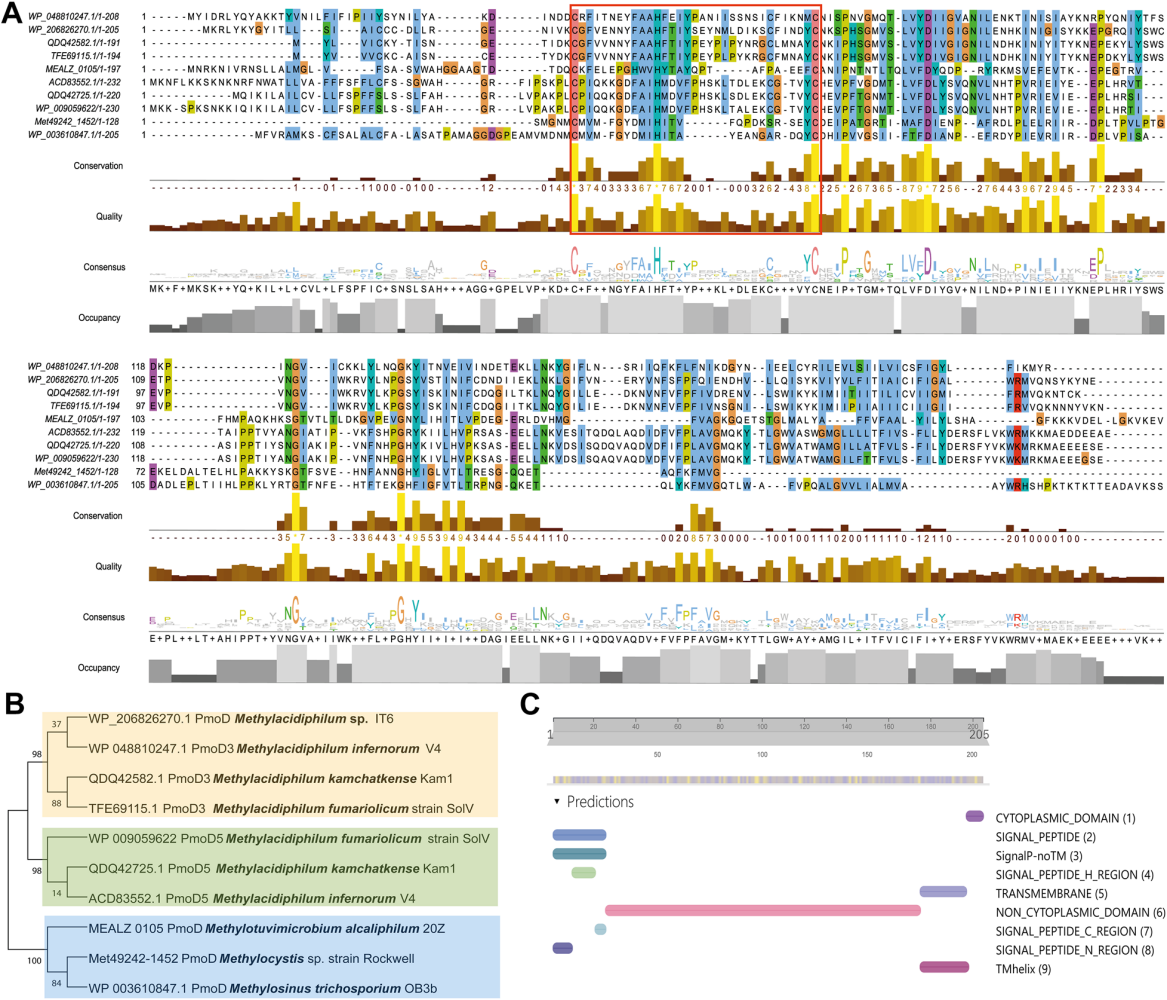
Bioinformatic analysis of the novel protein PmoD (GenBank accession number QSR88567) from *Methylacidiphilum* sp. IT6. **A** Multiple sequence alignment of the protein PmoD with reference protein sequences conducted using Clustal Omega [33] and viewed in Jalview program [34] with necessary annotations. The GenBank accession numbers of all reference sequences are displayed on the left of corresponding sequences. ACD83552.1: PmoD5 of *Methylacidiphilum infernorum* V4; WP_206826270.1: PmoD of *Methylacidiphilum* sp. IT6; MEALZ 0105: PmoD of *Methylotuvimicrobium alcaliphilum* 20Z; Met49242-1452: PmoD of *Methylocystis* sp. strain Rockwell; QDQ42582.1: PmoD3 of *M. kamchatkense* Kam1; QDQ42725.1: PmoD5 of *M. kamchatkense* Kam1; TFE69115.1: PmoD3 of *M. fumariolicum* strain SolV; WP_003610847.1: *pmoD* of *Methylosinus trichosporium* OB3b; WP_009059622: PmoD5 of *M. fumariolicum* strain SolV; WP_048810247.1: PmoD3 of *M. infernorum* V4. **B** Phylogenetic analysis of the novel protein PmoD with reference proteins. The phylogenetic tree was constructed using the Maximum Likelihood method with the Jones–Taylor–Thornton (JTT) matrix-based model and 500-replicate bootstrap [35]. **C** Predicted protein structures of PmoD of *Methylacidiphilum* sp. IT6 using InterProScan [25]

The BLASTP results of the protein PmoD showed that this protein matches the PmoD3 sequence of strain Kam1. The PmoD proteins of *M. kamchatkense* Kam1, together with those of two strains of *Methylacidiphilum* spp., *M. infernorum* V4, and *M. fumariolicum* strain SolV, were deployed for analysis [23]. Furthermore, Fisher and colleagues provided an insightful discussion on PmoD proteins encoded together with pMMO operons in proteobacterial methanotrophs [24]. Thus, in this study, to support the analysis of the novel protein PmoD, we collected reference PmoD protein sequences from three representative proteobacterial methanotrophs, *Methylocystis* sp. strain Rockwell, *M. alcaliphilum* 20Z, and *Methylosinus trichosporium* OB3b for later comparison and analysis [24]. The PmoD of strain IT6 contains 618 bp, encoding a 205-amino acid (aa) preprotein. Multiple alignments of this protein with reference sequences exposed a conserved region shared among all sequences shown in Fig. 1A. There are two conserved cysteine residues (Cys31 and Cys63) and one histidine residue (His42) between the PmoD sequences of *Methylacidiphilum* spp. and those of proteobacterial methanotrophs.

Phylogenetic tree analysis using the maximum likelihood method with the Jones–Taylor–Thornton (JTT) matrix-based model and 500-replicate bootstrap divided the PmoD sequences into three subgroups (Fig. 1B). The PmoD3 sequences of *M. infernorum* V4, *M. kamchatkense* Kam1, and *M. fumariolicum* strain SolV showed the highest likelihood with the novel protein PmoD of *Methylacidiphilum* sp. IT6. Moreover, this subgroup of PmoD3 sequences has a connection with the PmoD5 subgroups of the same three *Methylacidiphilum* spp. This implies their connection as homologs in the genus *Methylacidiphilum*. By contrast, the PmoD sequences of proteobacterial methanotrophs including *Methylocystis* sp. strain Rockwell, *M. alcaliphilum* 20Z, and *M. trichosporium* OB3b clustered in a separate subgroup, could be considered as an outgroup of the whole phylogenetic tree. The results suggested that the evolution of those PmoD proteins in the *Methylacidiphilum* genus, along with novel protein PmoD of *Methylacidiphilum* sp. IT6 has separated apart from those PmoD proteins in the proteobacterial counterpart.

We took a deeper look into the structure of the PmoD query protein using InterProScan to predict its domain as well as its family protein (Fig. 1C) [25]. InterProScan could not predict the family protein of PmoD because of the novelty of this PmoD protein family. However, the predicted structure of PmoD showed that it is a membrane protein with a C-terminal transmembrane helix from aa 176–198 and the signal peptide at the N-terminal constitutes the first 25 aa that exists in the PmoD5 homologs of all three strains of *Methylacidiphilum* spp., Kam1, SolV and V4 [23]. The protein PmoD was predicted to have a large non-cytoplasmic domain (aa 26–175) and a small cytoplasmic domain (aa 198–205) at the C-terminal. This non-cytoplasmic domain may locate in the periplasm as those PmoD proteins in proteobacterial methanotrophs [24].

### Construction of novel biocatalyst *Methylomonas* sp. DH-1 for acetone oxidation

**Fig. 2:**
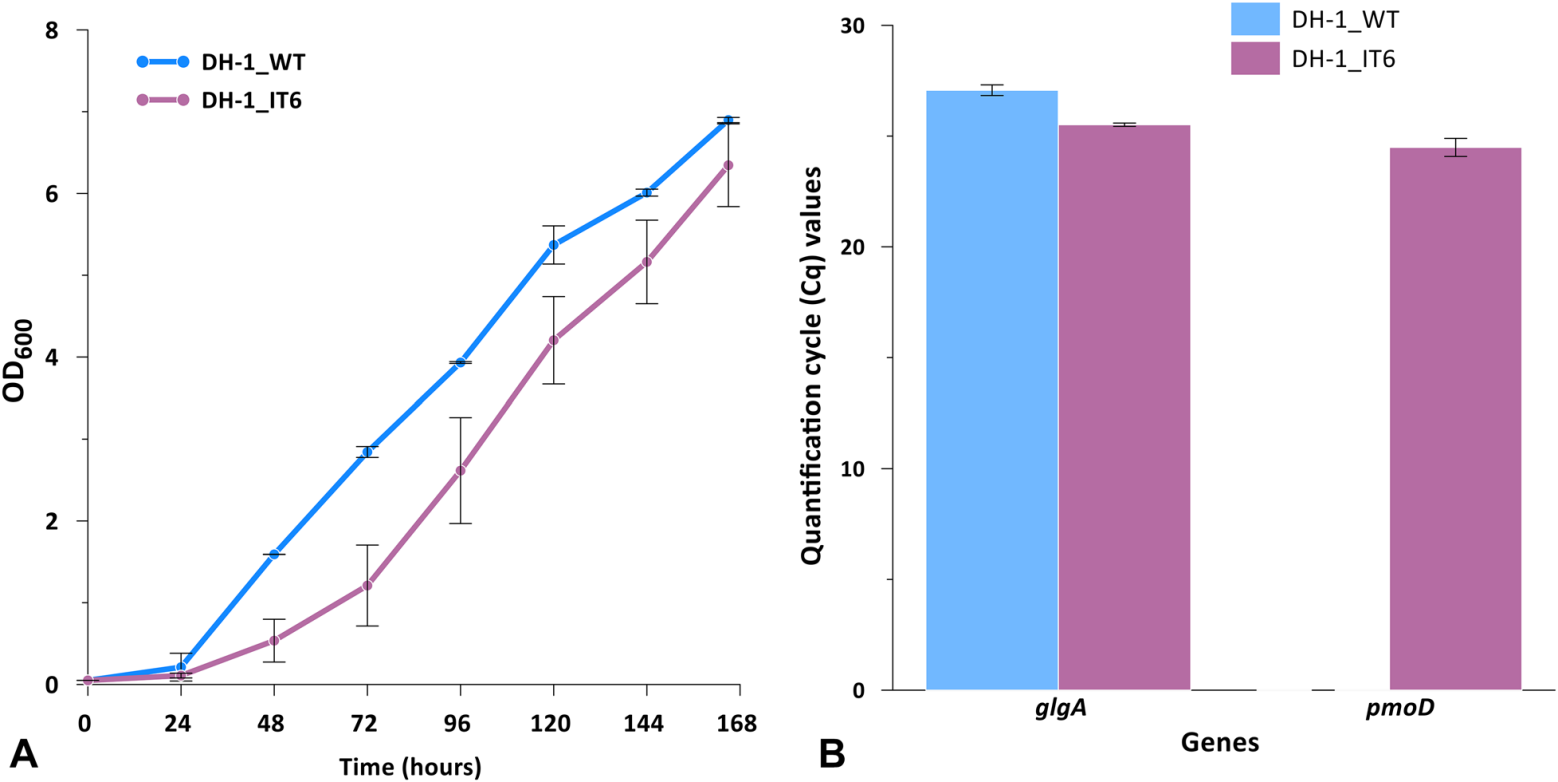
Growth rate (**A**) and the quantification cycle (Cq) values (**B**) of *Methylomonas* sp. DH-1 wild-type (DH-1_WT) and recombinant (DH-1_IT6) strains cultured in 30% (v/v) methane. Error bars represent the standard deviation. Three independent biological experiments were performed in triplicate, and one representative experiment was chosen for figure production

Although data from the previous study demonstrated the questionable role of novel protein PmoD for C3 substrate assimilation in strain IT6, the *Methylacidiphilum* genus is at its infancy with limited development of genetic tools for genetic modifications for the insightful investigation. Thus, heterologous expression of PmoD in other host strains to elucidate its involvement is a feasible approach. Furthermore, the heteroexpressed host can be deployed as a novel biocatalyst to achieve the green production of acetol from acetone. We integrated the *pmoD* into the *pmo* operon of *Methylomonas* sp. DH-1 to test the coupling activity of PmoD and pMMO to oxidize acetone. Linear DNA constructs were electroporated into the *Methylomonas* sp. DH-1 for the co-expression of *pmoD* with the *pmo* operon, with the constitutive expression controlled by the *E. coli* ς^70^-like promoter [26, 27]. The recombinant strain integrated with the *pmoD* DH-1_IT6 was verified using PCR. The strain showed a growth pattern similar to that of wild type with a slower rate, which indicated that the heterologous expression of *pmoD* interfered with cell growth (Fig. 2A).

Sodium dodecyl sulfate–polyacrylamide gel electrophoresis (SDS-PAGE) was conducted to check the expression of the PmoD protein in *Methylomonas* sp. DH-1 but the desired band could not be detected (data not shown). This could be due to the low protein expression of *Methylomonas* sp. DH-1, consistent with our previous study [20]. To identify the expression of PmoD in the DH-1_IT6 recombinant strain, the total RNA of *Methylomonas* sp. DH-1 wild-type (DH-1_WT) and DH-1_IT6 recombinant strains were used for RT-qPCR to check the expression of PmoD at the transcriptional level. Water was used as a negative control and the glycogen synthase (*glgA*) as a reference gene. The quantification cycle (Cq) values of DH-1 wild-type and DH-1_IT6 recombinant cultured in 30% methane are shown in Fig. 2B. The RNA levels of *glgA* were similar in reactions of wild type and recombinant with the Cq above 25. In the case of PmoD, there was no RNA trace detected in DH-1 wild type, while the Cq values of DH-1_IT6 recombinant were similar to those of the reference gene *glgA*. These results demonstrated the expression of PmoD in the DH-1_IT6 recombinant strain at the transcriptional level.

### Bioconversion of acetol from acetone in whole-cell biocatalyst

Hur and colleagues demonstrated the bioconversion of propane to acetone in *Methylomonas* sp. DH-1 [17]. Propane was first oxidized into 2-propanol through pMMO and then into acetone by alcohol dehydrogenases using *Methylomonas* sp. DH-1 as a whole-cell biocatalyst. Thus, in this study, we employed recombinant strain DH-1_IT6 as a whole-cell biocatalyst to demonstrate the coupling activity of this novel protein with the endogenous pMMO of *Methylomonas* sp. DH-1 by evaluating the acetol production.

**Fig. 3:**
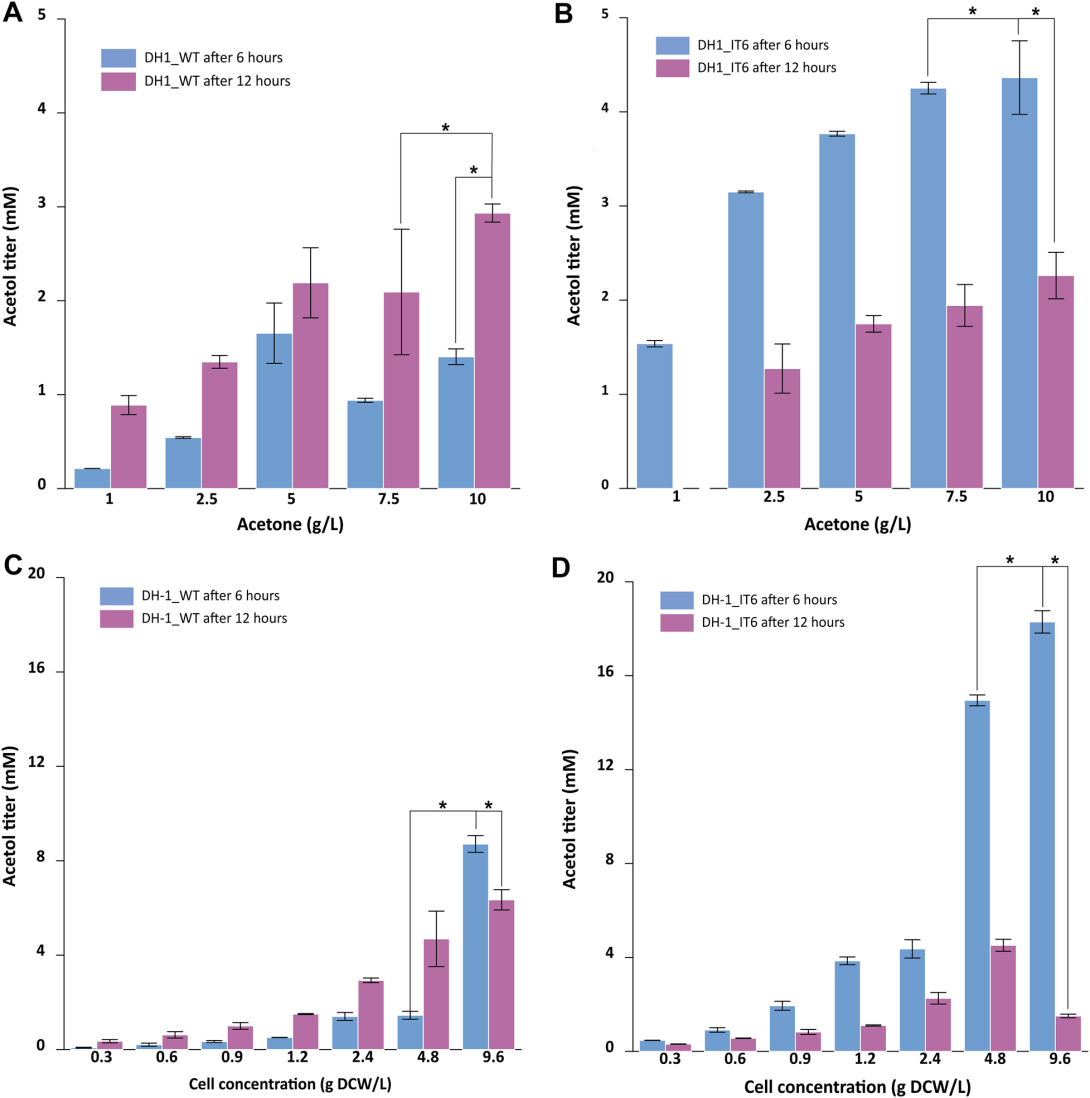
Bioconversion of acetone to acetol in the whole-cell biocatalyst. The acetol titers of *Methylomonas* sp. DH-1 wild-type (DH-1_WT) and recombinant (DH-1_IT6) at different incubation times (6 h and 12 h) under various acetone concentrations (1, 2.5, 5, 7.5 and 10 g/L) (**A**, **B**) and cell mass concentrations (0.3, 0.6, 0.9, 1.2, 2.4, 4.8 and 9.6 gDCW/L) (**C**, **D**). Error bars represent the standard deviation. Three independent experiments were performed in triplicate and one representative experiment was chosen for figure production. *, *P* < 0.05, significant differences were noted with two-way ANOVA statistical analysis

Acetone production from oxidation of 2-propanol or propane was studied well in a previous study. However, under optimal conditions, apart from acetone, the trace of acetol was not detected [17]. Thus, we decided to use acetone as a direct substrate for conversion to acetol in this study. The experiment was conducted using a cell mass concentration of 2.4 g dry cell weight (DCW)/L with acetone concentrations of 1, 2.5, 5, 7.5, and 10 g/L (Fig. 3A, B). Whole-cell reactions were incubated and sampled after 6 and 12 h. Other conditions were similar to those described in previous studies [17, 22]. Acetol production in *Methylomonas* sp. DH-1 wild type increased with incubation time, and the acetol titers of DH-1_IT6 in all reactions were the highest at the 6-h mark but eventually decreased. The highest acetol titers achieved were 2.934 mM for DH-1_WT after 12-h incubation and 4.364 mM for DH-1_IT6 after 6-h incubation, which is nearly 1.5 times higher. The maximum conversion rates of DH-1_WT and DH-1_IT6 were 0.244 mM/h after 12 h and 0.727 mM/h after 6 h, respectively.

In addition to optimizing substrate concentrations, we optimized the bacterial cell concentrations in the whole-cell biocatalyst reaction (Fig. 3C, D). In terms of DH-1-WT reactions, the changes in acetol titers shared similar patterns with the acetone concentration optimization experiment (Fig. 3C). The titers of acetol increased in two dimensions: incubation time and cell concentration. In the range of 0.3 to 4.8 gDCW/L, the titers of acetol increased corresponding with incubation time but were still lower than 5 mM (4.689 mM being the highest). The reactions with a cell concentration of 9.6 gDCW/L showed a different trend with the highest titer of 8.709 mM (645.13 mg/L) obtained after 6-h incubation. In terms of the reactions of DH-1_IT6, two noticeable results were observed in the reactions with cell concentrations of 4.8 and 9.6 gDCW/L. The maximum accumulated acetol was 18.291 mM (1.35 g/L), with the specific productivity was 0.317 mmol/g cell/h, which were achieved with a cell concentration of 9.6 gDCW/L and acetone concentration of 10 g/L.

### Bioconversion of acetone to acetol in batch cultivation

From the results of the whole-cell biocatalyst experiment, we further studied the bioconversion of acetone to acetol under batch conditions. A previous study on *Methylocella silvestris* BL2 demonstrated the capacity of cytochrome P450 in the conversion of acetone into acetol [11]. Thus, in this study, we constructed a cytochrome P450_deleted mutant (DH-1_ΔP450) to validate its capacity for acetone conversion in *Methylomonas* sp. DH-1 wild type (see Additional file 1: Fig. S4 for related information).

**Fig. 4:**
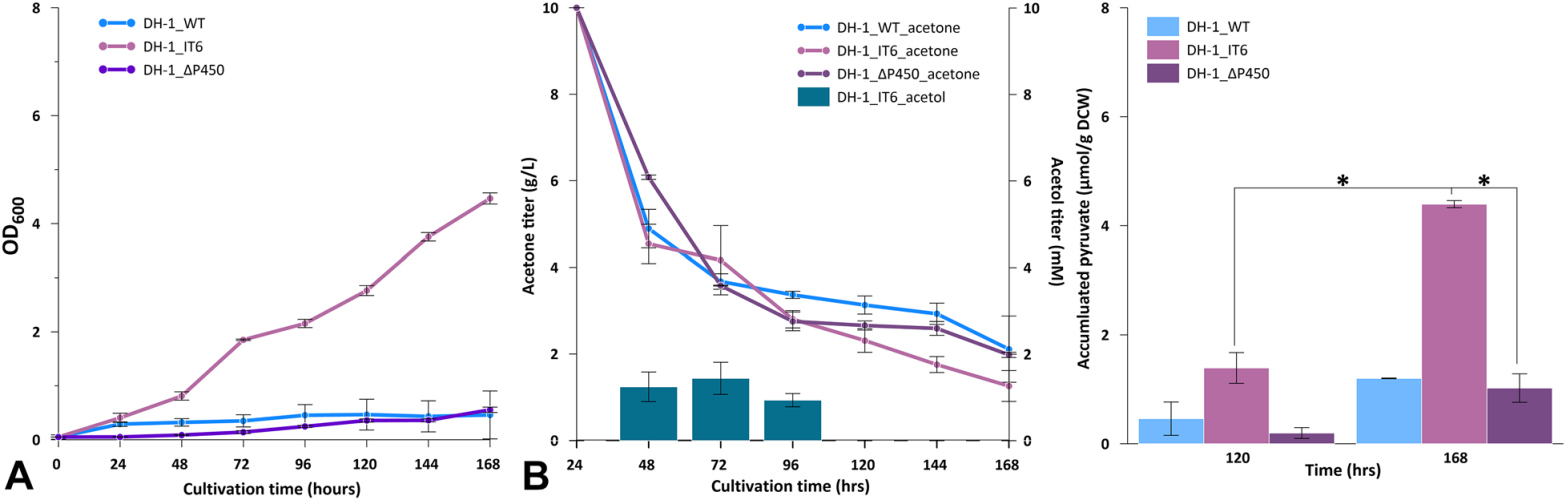
Bioconversion of acetone to acetol under batch conditions. **A** Growth rate of *Methylomonas* sp. DH-1 wild-type (DH-1_WT) and recombinant (DH-1_IT6 and DH-1_ΔP450) under the cultivation conditions of 30% (v/v) methane with the addition of acetone to the titer of 10 g/L after the first 24-h cultivation. **B** Titer of acetone and acetol of DH-1_WT, DH-1_IT6, and DH-1_ΔP450 under cultivation of 30% (v/v) methane with the addition of acetone to the titer of 10 g/L after 24 h. **C** Accumulation of intracellular pyruvate extracted from cell mass of DH-1_WT, DH-1_IT6, and DH-1_ΔP450 at 120 and 168 h timepoints. Error bars represent the standard deviation. Three independent biological experiments were performed in triplicate and one representative experiment was chosen for figure production. *, *P* < 0.05, significant differences were noted with two-way ANOVA statistical analysis

To elucidate the possibility of acetone assimilation, we cultivated *Methylomonas* sp. DH-1 wild-type and recombinant strains used only acetone as the carbon source, but their growth was prohibited. Thus, we cultured in batch conditions using methane as the carbon source along with the acetone concentration of 10 g/L. To avoid the growth inhibition by acetone because of low-cell concentration with optical density (OD_600_) ~ 0.05, we supplied acetone to the culture after 24-h cultivation. The growth rates of both *Methylomonas* sp. DH-1 wild-type and recombinant strains were recorded, and the supernatants were collected for gas chromatography (GC) analysis. The growth rates of both wild-type and recombinant strains demonstrated a depressed growth because of acetone (Fig. 4A). However, the growth of *Methylomonas* sp. DH-1 wild-type and DH-1_ ΔP450 was significantly inhibited with an OD_600_ of approximately 0.5, whereas the recombinant strain DH-1_IT6 was able to surpass the inhibitory effects of acetone and grew up to the OD_600_ of 4.46.

The amount of remaining acetone and acetol produced in wild-type and recombinant strains are shown in Fig. 4B. There was a significant drop in the acetone titer after the first 24 h in both wild-type and recombinant treatments. A significant amount of acetone (nearly 50% of the initial titer) was absorbed, considering that an amount of acetone was evaporated (see Additional file 1: Figure S3). Among wild-type and recombinant treatments, acetol was detected only in DH-1_IT6 recombinant cultures from 48 to 96 h with the highest titer of 1.443 mM obtained at the 72-h mark.

In terms of the intracellular pyruvate accumulated in wild-type and recombinant strains, the amount of pyruvate was evaluated at two timepoints, 120 and 168 h. The accumulation of pyruvate inside the treatments of DH-1_IT6 was the highest among the three strains at two evaluation timepoints. The highest amount of DH-1_IT6 is at 168 h with 4.394 µmol pyruvate/gDCW, which is 3.65-fold and 4.3-fold higher than those of DH-1_WT and DH-1_ ΔP450.

## Discussion

From the transcriptomic data of the previous study [12], the novel protein PmoD of *Methylacidiphilum* sp. IT6 raised the inquiry of its involvement in the acetone oxidation capacity of the PmoCAB3, a pMMO homolog. Thus, in this study, we deployed various bioinformatics tools to achieve some preliminary elucidations of this protein and also heteroexpressed this protein in the methanotrophic host *Methylomonas* sp. DH-1 to evaluate the coupling activity of this protein PmoD with endogenous pMMO in acetol production.

The multiple alignment results revealed a conserved region of this novel protein PmoD with other reference PmoD sequences, composed of two cysteines (Cys31 and Cys63) and one histidine (His42) residue. As described in a previous study, two strictly conserved Cys residues of the surface-exposed region located near the N-terminus of PmoD proteins were suggested to play the main role in the copper-binding of proteobacterial methanotrophs, where each PmoD monomer contributes a Cys ligand to form bridging thiolates at the copper-binding site [24]. However, at the position of the conserved methionine residue in the PmoD sequences of proteobacterial methanotrophs, it is substituted by alanine residue, which is conserved in the novel protein PmoD of *Methylacidiphilum* sp. IT6, and other PmoD homologs (PmoD3 and PmoD5) of *M. infernorum* V4 and *M. fumariolicum* strain SolV. This replacement has been shown to eliminate the formation of copper-binding sites in proteobacterial methanotrophs [24], which raises the hypothesis about the function of the novel protein PmoD of strain IT6 and other PmoD proteins of the *Methylacidiphilum* genus. Furthermore, the phylogenetic tree analysis using MEGA X and structural prediction using InterProScan were to get further insights into the evolutionary and structural characteristics of the novel protein PmoD. The predicted structure of PmoD suggested it as the membrane protein which is similar to the structures of those PmoD sequences in proteobacterial methanotrophs. Still, the distant connection of the PmoD of *Methylacidiphilum* sp. IT6 with those of proteobacterial methanotrophs demonstrated using the phylogenetic tree analysis, together with the results from the multiple alignment analysis, raised an intriguing question on the functions of this protein, rather than the copper-binding capacity.

To date, there have been no reports on the ability of *Methylomonas* sp. DH-1 to convert acetone to acetol under normal culture conditions. As the whole-cell biocatalyst using propane and 2-propanol as substrates, acetone was the sole product detected [17]. Thus, by integrating the novel protein PmoD of strain *Methylacidiphilum* sp. IT6 into the methanotrophic *Methylomonas* sp. DH-1, we conducted the whole-cell biocatalyst assays to evaluate the coupling activity of PmoD with pMMO of *Methylomonas* sp. DH-1 to enable the production of acetol from acetone. In this experiment, the reactions of DH-1_WT showed traces of acetol produced from acetone which was unexpected at first. Although *Methylomonas* sp. DH-1 contains cytochrome P450, an enzyme proved to have the capacity to convert acetone to acetol [10, 11], the gene expression level in *Methylomonas* sp. DH-1 was low with 322 RPKM (reads per kilobase of transcript per million reads mapped) in comparison with 953,554 RPKM of *pmoC* subunit of pMMO under methane cultured conditions [17]. Thus, a high concentration of acetone of 10 g/L, in this case, could trigger cytochrome P450 activity in *Methylomonas* sp. DH-1 to convert acetone to acetol. The mutant DH-1_ΔP450 was deployed in the whole-cell biocatalyst conditions to testify our hypothesis. Under the optimal conditions, although the acetone concentration was reduced, there is no trace of acetol detected in these reactions (data not shown), which reinforces for our conclusion on the role of cytochrome P450 in acetol production. This study is the first to demonstrate the acetone oxidation capacity in type I methanotroph. After the optimization of acetone and cell mass concentrations, the acetol titer achieved in whole-cell biocatalyst is 18.291 mM (1.35 g/L) which is much higher than that in the *E. coli* expressing MimABCD cluster (1.02 mM under whole-cell conditions) [6] or even comparable with the result in METabolix Explorer’s patent (1.63 g/L acetol produced from glucose in *E. coli*) [2]. These results demonstrated that the novel protein PmoD was successfully expressed in the *Methylomonas* sp. DH-1, followed by an enhancement in the oxidation of acetone to acetol.

The recent discovery of the acetone oxidation obtained in the copper membrane methane monooxygenase homolog PmoCAB3 in *Methylacidiphilum* sp. IT6 [12]. Considering the similar reaction mechanism as PmoCAB3 a homolog of pMMO, the role of novel protein PmoD was evaluated in this study by co-expressing with the *pmo* operon of *Methylomonas* sp. DH-1 led to a boost in acetol production from acetone. Together with the bioinformatics analyses, we proposed that PmoD acts as a transport protein to catch and deliver acetone to the active site of the complex to allow the oxidation of acetone. This could explain its similar expression patterns together with that of the proposed acetone monooxygenase in strain *Methylacidiphilum* sp. IT6. However, further experiments are needed to elucidate the activity of PmoD and its specific mechanism in assisting the oxidation reaction of pMMO complexes.

Although we recorded the conversion of acetone to acetol under the whole-cell biocatalyst conditions in DH-1 wild type, the effects of acetone on living cells under batch conditions may be more complicated, and the activity of cytochrome P450 may not be sufficient to detoxify a high acetone concentration. Furthermore, the inhibition of cell growth by acetone could result from various effects on cellular physiology. The adverse effects and the penetration mechanism of acetone into the bacterial cell membranes were elucidated in previous studies [28, 29]. The acetone effects on the structure and dynamics of bilayer lipid membranes were intensively studied using molecular dynamics simulations. The study demonstrated a favorable tendency of acetone passively penetrating to the hydrophobic regions of the membrane with the potential energy of − 3.6 kcal/mol. A high concentration of acetone caused adverse effects on the phospholipid membrane organization and fluidity [28]. Another study showed that the growth of bacterial strains was hindered by the adverse effects of organic solvent, specifically acetone, on the fluidity of cell membranes, thereby affecting the supply of nutrients to the cell [29]. This led to the hypothesis that the DH-1_IT6 recombinant strain could bypass the inhibitory effects of acetone by converting acetone into other substrates, such as acetol, which is less toxic to bacterial cells. Furthermore, the acetol produced was only detected during the first 3 days after the addition of acetone. Subsequently, although acetone titer continued to decrease to the lowest titer of 1.259 g/L at 168 h, there was no trace of acetol detected in any reactions of both wild-type and recombinant strains. In comparison with the whole-cell biocatalyst experiment, the highest produced acetol titer in batch conditions was nearly 12-fold less (1.443 mM in comparison with 18.291 mM). These different titers should be in consideration of the differences in cell mass concentrations, sampling times, and cultivation conditions.

**Fig. 5:**
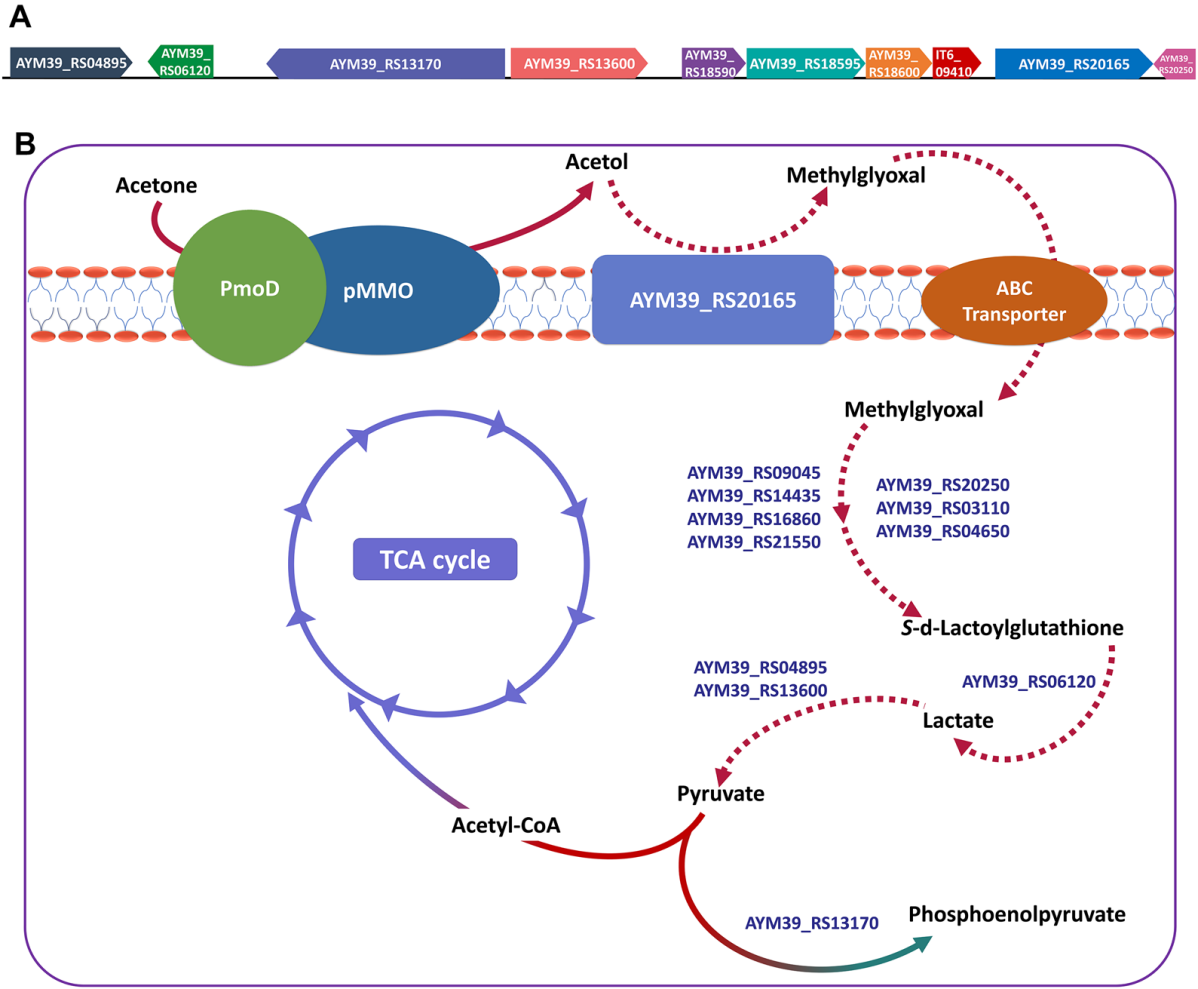
Proposed acetone assimilation pathway in *Methylomonas* sp. DH-1. **A** Organization of related genes in the proposed acetone assimilation pathway on the chromosome of the methanotrophic *Methylomonas* sp. DH-1. **B** Particulate methane monooxygenase (pMMO; AYM39_RS18590, AYM39_RS18595, and AYM39_RS18600) co-expressed with novel PmoD (IT6_09410) of *Methylacidiphilum* sp. IT6 oxidizes acetone to acetol, which could be further oxidized to methylglyoxal by GMC oxidoreductase (AYM39_RS20165). Passive diffusion or active transportation via transporter protein (ABC transporter) may be responsible for the methylglyoxal transportation into the cytoplasm. Seven candidate genes (AYM39_RS20250, AYM39_RS03110, AYM39_RS04650, AYM39_RS09045, AYM39_RS14435, AYM39_RS16860, AYM39_RS21550) annotated as glyoxalase enzymes may convert methylglyoxal into S-lactoylglutathione, which can be further converted into lactate by hydroxyacylglutathione hydrolase (AYM39_RS06120) and then to pyruvate by putative lactate dehydrogenase (AYM39_RS04895, AYM39_RS13600). The generated pyruvate can be either converted to acetyl-CoA to enter the TCA cycle or to phosphoenolpyruvate (PEP) by PEP-synthase (AYM39_RS13170). The related genes are shown using their locus tags obtained from the genome of *Methylomonas* sp. DH-1 (GenBank accession number NZ_CP014360). The related reactions of the proposed pathway are demonstrated by dashed arrows

The disappearance of the acetol titer in the following cultivation days could have two possible explanations. The decrease in the acetone titer may have led to a decrease of the acetol titer produced to untraceable levels. However, we observed a decrease in the acetol titer before its disappearance which also indicates the possibility that the acetol converted from acetone to detoxify the inhibitory effects of acetone was further assimilated by *Methylomonas* sp. DH-1. The hypothesis was reinforced with the data obtained from intracellular pyruvate accumulation. While being cultivated with methane and acetone, the recombinant strains DH-1_IT6 showed a significantly higher amount of pyruvate accumulated inside the cells (4.39 µmol/gDCW in DH-1_IT6 compared to 1.20 µmol/gDCW in DH-1_WT or 1.02 µmol/gDCW in DH-1_ ΔP450), which implies for their ability to assimilate acetol into pyruvate for further metabolism. As mentioned in a previous study, acetol could be converted to methylglyoxal by acetol dehydrogenase, which could be further converted into pyruvate [30]. The proposed pathway for acetone assimilation in strain IT6 was discussed in a previous study based on the genomic and transcriptomic data [12]. Their proposed pathway was based on the one described in *Methylocella silvestris* BL2 [11], in which glucose–methanol–choline (GMC) oxidoreductase could convert acetol to methylglyoxal, which was further converted to lactate by vicinal oxygen chelate proteins (glyoxalases I and II). Further conversion of lactate to pyruvate, an important intermediate for cellular metabolism, was catalyzed by Fe–S- and FAD-binding motif-containing proteins, which could compose a protein with similar activity as the FAD-dependent lactate dehydrogenase [12, 31]. About *Methylomonas* sp. DH-1 genome, all necessary component proteins of the discussed pathway were able to be identified (Fig. 5), especially genes coding for 7 candidate glyoxalases exist in the genome of *Methylomonas* sp. DH-1 (See the Additional file 1: Table S3). Although a possible pathway for the assimilation of acetone or even propane exists in *Methylomonas* sp. DH-1, no reports on the growth of *Methylomonas* sp. DH-1 on these C3 substrates was reported. This could be due to the toxicity of the intermediates of the pathway, such as acetone and lactate, which could suppress the growth of *Methylomonas* sp. DH-1 [21]. With the expression of PmoD and coupling activity of PmoD and pMMO for acetone oxidation, although *Methylomonas* sp. DH-1 cannot solely assimilate for growth, propane or other C3 intermediates (2-propanol and acetone) could be metabolized together with methane as an auxiliary energy source for *Methylomonas* sp. DH-1 metabolism via acetol conversion. However, further studies are required to deeply scrutinize this proposed pathway and its potential for future applications.

## Conclusion

In this study, the PmoD from *Methylacidiphilum* sp. IT6 was elucidated as a membrane protein with the proposed function as a transport protein that captures and delivers acetone to the pMMO for oxidation. The coupling activity of heteroexpressed PmoD with the endogenous pMMO of *Methylomonas* sp. DH-1 led to a boost in the acetone oxidation to acetol in whole-cell biocatalyst assays with the highest titer of 18.291 mM (1.35 g/L) under optimal conditions. This result illustrates the potential of whole-cell biocatalyst *Methylomonas* sp. DH-1 in acetol production. Furthermore, the innate capacity of *Methylomonas* sp. DH-1 wild type to oxidize acetone to acetol as a whole-cell biocatalyst has been demonstrated, which is an unprecedented report in type I methanotrophic bacteria. From the data achieved in batch cultivation conditions and the reference with previous publications, we proposed a novel assimilation pathway of acetone or propane via acetol as the key intermediates. Once validated, this assimilation pathway can extend the metabolic flexibility of *Methylomonas* sp. DH-1.

## Methods

### Media and culture conditions

The strains used in this study are listed (see Additional file 1: Table S1). *Methylomonas* sp. DH-1 was cultured in a nitrate mineral salt medium containing 10 μM CuSO_4_ under the culture conditions described in a previous study [17]. A 50-mL culture of the *Methylomonas* sp. DH-1 recombinant strain was inoculated into pre-cultured cells at an OD_600_ of 0.05 for growth rate experiments. Zeocin (Zeo) was used at a final concentration of 30 μg/mL to select recombinant *Methylomonas* sp. DH-1. *Methylacidiphilum* sp. IT6 was kindly provided by Prof. Sung-Keun Rhee from Chungbuk National University (personal communication).

### Bioinformatics analyses of the novel protein PmoD in *Methylacidiphilum* sp. IT6

The protein sequence of PmoD (GenBank accession number QSR88567) was obtained from *Methylacidiphilum* sp. IT6 genome (GenBank accession number CP065957). The protein sequence of PmoD was screened by BLASTP using the NCBI non-redundant protein sequence database to search for potential reference protein sequences [32]. Based on the BLASTP results, we collected more related protein sequences from two publications, Fisher et al*.* [24] and Kruse et al*.* [23]. All sequences were aligned using Clustal Omega [33] and viewed using the Jalview program with necessary annotations [34]. The query cover and percent identity of reference sequences in comparison with query sequence PmoD were achieved using BLASTP. MEGA X version 10.0.5 was used for the phylogenetic analysis [35] using the maximum likelihood method with the JTT matrix-based model and 500-replicate bootstrap. All gaps and missing data were deleted using the complete deletion method. InterProScan was used to predict the domains of the PmoD sequence [25]. Other subunits of *Methylacidiphilum* sp. IT6 as PmoC3, PmoA3, and PmoB3 were also analyzed using multiple sequence alignment with Clustal Omega, and BLASTP.

### Genetic manipulations

The genomic DNA of *Methylacidiphilum* sp. IT6 and *Methylomonas* sp. DH-1 was isolated, and molecular engineering techniques were conducted as described in an earlier study [36].

Gene integration constructs were assembled from PCR products which were then electroporated into *Methylomonas* sp. DH-1 as described earlier [20]. Linear DNA fragments for genetic integration were constructed as previously described [37]. In brief, a DNA construct containing the required coding genes and Zeo resistance gene was flanked by two flanking regions of the target integrating position, right after the last gene-coding sequence of the *pmo* operon. In this study, the construct contained the *pmoD* gene of *Methylacidiphilum* sp. IT6 and the Zeo resistance gene were integrated next to the *pmo* operon of *Methylomonas* sp. DH-1 for the co-expression of *pmoD* together with the *pmo* operon. The mutants were screened by culturing on media containing Zeo, and the verification of successful integration was determined using PCR with a pair of primers of the *pmoD* gene. Vector pCM184-p450 was constructed for developing the DH-1 mutant (DH-1_ΔP450). The successful deletion mutants were screened by culturing on media containing Kanamycin (Km) and verified by PCR using gene-specific primer pair (see Additional file 1: Figure S4A).

Cell lysates of wild-type and recombinant strains of *Methylomonas* sp. DH-1 were also prepared according to the protocol described on the European Molecular Biology Laboratory website [38]. The lysates were electrophoresed using 13% SDS-PAGE and stained using the Pierce™ Silver Stain kit (Thermo Fisher Scientific, USA). The primers used for genetic construction are listed (see the Additional file 1: Table S2).

### Total RNA isolation and RT-qPCR

All cultures were grown in biological duplicates for subsequent RNA extraction and RT-PCR. RNA extraction and RT-qPCR were conducted as described in the previous studies [36, 39]. The Cq value for each gene was determined and the *glgA* was used as a reference. The primer sequences used for RT-qPCR are also listed (see Additional file 1, Table S2).

### The whole-cell biocatalyst for acetone bioconversion

The whole-cell biocatalyst experiment was performed as previously described [17]. To optimize the reaction parameters, acetone concentrations of 1, 2.5, 5, 7.5, and 10 g/L were supplied in the whole-cell reactions. Furthermore, a range of different cell mass concentrations (0.3, 0.6, 0.9, 1.2, 2.4, 4.8, and 9.6 gDCW/L) was tested with the optimal acetone concentration.

### Bioconversion of acetone to acetol in batch cultivation

Wild-type and recombinant *Methylomonas* sp. DH-1 strains were cultured in biological duplicates with an OD_600_ of 0.05, under 30% (v/v) methane. After 24 h, acetone was added at a concentration of 10 g/L for all treatments. The headspace was refreshed, and media cultures were collected daily. The culture samples were centrifuged at 10,000 X *g* for 10 min, and the supernatants were collected and filtered through a 0.2 µm membrane before injection into the GC. Furthermore, the intracellular pyruvate accumulation was evaluated at two timepoints, 120 and 168 h. The extraction was conducted with perchloric acid 6% following described procedure [40].

### Analysis using gas chromatography

The supernatant was quantified using GC (Younglin, 6500GC) equipped with an HP-Innowax column (30 m × 0.53 mm inner diameter) and a flame ionization detector. The injection was conducted in splitless mode with 1 µL samples. The oven temperature was maintained at 100 ℃ for 3 min and then increased 10 °C/min to 215 ℃. The extracted pyruvate was measured by HPLC (Jasco Co., Japan) as described in a previous study [20]. Three independent biological experiments were performed in triplicate, and one representative experiment was chosen for figure production. Statistical analysis of variance (ANOVA) was employed to compare mean values, with * = *P* < 0.05 considered to be statistically significant.

## Supplementary Information

**Additional file 1: Table S1.** All bacteria strains and plasmids used in this study. **Table S2.** Primers used in this study. **Table S3.** Identification of proteins of the acetone assimilation gene cluster in *Methylacidiphilum* sp. IT6 and *Methylomonas* sp. DH-1 with the exclusion of particulate methane monooxygenase and novel PmoD protein. **Figure S1.** Multiple sequence alignment by Clustal Omega and BLASTP results of PmoA3 (A), PmoB3 (B) and PmoC3 (C) of *Methylacidophilum* sp. IT6 with corresponding proteins sequences of *Methylacidiphilum kamchatkense* Kam1, *Methylotuvimicrobium alcaliphilum* 20Z, and *Methylomonas* sp. DH-1. **Figure S2.** PCR results of *Methylomonas* sp. DH-1 wild-type (DH-1_WT) and recombinant (DH-1_IT6). A) Confirmation of the integration of *pmoD* of *Methylacidophilum* sp. IT6 into *Methylomonas* sp. DH-1 using the pair of primers to amplify the *pmoD*; B) Electrophoresis results of RT-PCR products to test the expression of the PmoD in *Methylomonas* sp. DH-1 recombinant. **Figure S3.** Acetone titers remained in 50 ml of NMS medium in the 500-ml baffled-flask sealed with a screw cap incubated at 30 °C and 250 rpm. The headspace was supplied with 30% (v/v) methane by a gas-tight syringe. The headspace was refreshed every day. Error bars represent the standard deviation. Three independent biological experiments were performed in triplicate and one representative experiment was chosen for figure production. **Figure S4.** PCR results and growth rate of cytochrome P450-deleted *Methylomonas* sp. DH-1 (DH-1_ΔP450) A) Confirmation of the deletion of cytochrome P450 in *Methylomonas* sp. DH-1 using the pair of primers to amplify the cytochrome P450 coding sequence; B) Electrophoresis results of RT-PCR products to test the expression of the PmoD in *Methylomonas* sp. DH-1 recombinant. Growth rate (A) of *Methylomonas* sp. DH-1 wild-type (DH-1_WT) and recombinant (DH-1_ΔP450) strains cultured in 30% (v/v) methane. Error bars represent the standard deviation. Three independent biological experiments were performed in triplicate, and one representative experiment was chosen for figure production.

## Data Availability

The data sets used and/or analyzed during the current study are available from the corresponding author on reasonable request.

## Abbreviations

aa: Amino acid
Cq: Quantification cycle
DH-1_WT: DH-1 wild type
DH-1_IT6: The PmoD integrated DH-1 recombinant
DH-1_ ΔP450: The cytochrome P450-deleted DH-1 mutant
GC: Gas chromatograph
*glgA*: Glycogen synthase
GMC: Glucose–methanol–choline
pMMO: Particulate methane monooxygenase
PmoCAB3: Proposed acetone monooxygenase
SDS-PAGE: Sodium dodecyl sulfate–polyacrylamide gel electrophoresis
