# Supplementary material for: Boosting the acetol production in methanotrophic biocatalyst Methylomonas sp. DH-1 by the coupling activity of heteroexpressed novel protein PmoD with endogenous particulate methane monooxygenase
Source: Biotechnol Biofuels Bioprod. 2022 Jan 17;15:7. doi: 10.1186/s13068-022-02105-1 (PMC8764830; doi:10.1186/s13068-022-02105-1)
Supplement: Supplementary file 1 — Additional file 1: Table S1. All bacteria strains and plasmids used in this study. Table S2. Primers used in this study. Table S3. Identification of proteins of the acetone assimilation gene cluster in Methylacidiphilum sp. IT6 and Methylomonas sp. DH-1 with the exclusion of particulate methane monooxygenase and novel PmoD protein. Figure S1. Multiple sequence alignment by Clustal Omega and BLASTP results of PmoA3 (A), PmoB3 (B) and PmoC3 (C) of Methylacidophilum sp. IT6 with corresponding proteins sequences of Methylacidiphilum kamchatkense Kam1, Methylotuvimicrobium alcaliphilum 20Z, and Methylomonas sp. DH-1. Figure S2. PCR results of Methylomonas sp. DH-1 wild-type (DH-1_WT) and recombinant (DH-1_IT6). A) Confirmation of the integration of pmoD of Methylacidophilum sp. IT6 into Methylomonas sp. DH-1 using the pair of primers to amplify the pmoD; B) Electrophoresis results of RT-PCR products to test the expression of the PmoD in Methylomonas sp. DH-1 recombinant. Figure S3. Acetone titers remained in 50 ml of NMS medium in the 500-ml baffled-flask sealed with a screw cap incubated at 30 °C and 250 rpm. The headspace was supplied with 30% (v/v) methane by a gas-tight syringe. The headspace was refreshed every day. Error bars represent the standard deviation. Three independent biological experiments were performed in triplicate and one representative experiment was chosen for figure production. Figure S4. PCR results and growth rate of cytochrome P450-deleted Methylomonas sp. DH-1 (DH-1_ΔP450) A) Confirmation of the deletion of cytochrome P450 in Methylomonas sp. DH-1 using the pair of primers to amplify the cytochrome P450 coding sequence; B) Electrophoresis results of RT-PCR products to test the expression of the PmoD in Methylomonas sp. DH-1 recombinant. Growth rate (A) of Methylomonas sp. DH-1 wild-type (DH-1_WT) and recombinant (DH-1_ΔP450) strains cultured in 30% (v/v) methane. Error bars represent the standard deviation. Three independent biological experime [file 13068_2022_2105_MOESM1_ESM.docx]

Additional file 1

**Boosting the acetol production in engineered methanotrophic biocatalyst *Methylomonas* sp. DH-1 by the coupling activity of heteroexpressed novel protein PmoD with endogenous particulate methane monooxygenase**

Tin Hoang Trung Chau^1^, Anh Duc Nguyen^1^ and Eun Yeol Lee^1,^*

^1^ Department of Chemical Engineering (Integrated Engineering), Kyung Hee University, Yongin-si, Gyeonggi-do 17104, South Korea

^*^**Corresponding author:**

Professor Eun Yeol Lee

Email: eunylee@khu.ac.kr, Phone: +82-31-201-3839 , Fax: +82-31-204-8114

**Table S1.** All bacteria strains used in this study

| **Strain** | **Characteristic(s)** | **Reference** |
| --- | --- | --- |
| *Methylacidphilum* sp. IT6 | Wild-type strain | 1 |
| *Methylomonas* sp. DH-1 | Wild-type strain | 2 |
| DH-1_IT6 | *Methylomonas* sp. DH-1 integrated the fragment containing pmoD of *Methylacidiphilum* sp. IT6 and Zeocin resistant gene next to the *pmo* operon | This study |
| DH-1_ΔP450 | *Methylomonas* sp. DH-1 with ΔP450 | This study |
| **Plasmids** | **Characteristic(s)** | **Reference** |
| pCM184 | Deletion/Integration vector, KmR | 3 |
| pCM184_p450 | pCm184, cytochrome P450 deletion vector | This study |

**Table S2.** Primers used in this study

| **Primer** | **Sequence** | **Description** |
| --- | --- | --- |
| Fwd_F1 flanking region | CTGGCACCCAAGGAAGAAATTCG | For amplifying F1 flanking region from *Methylomonas* sp. DH-1 genomic sequence |
| Rev_F1 flanking region | TAGCTGTTTCCTGTGTGAAGGGTTTAGTTGAAAACAGACCTATTC |  |
| Fwd_*pmoD* | CAACTAAACCCTTCACACAGGAAACAGCTATGAAAAGACTATATAAATATGGATATATAAC | For amplifying *pmoD* from *Methylacidiphilum* sp. IT6 genomic sequence with overhang sequences for ligation |
| Rev_*pmoD* | CAACAGCTCATTTCAGAGTTATTCGTTATACTTATAACTATTCTGAACCATTC |  |
| Fwd_ZeocinR | CTCTGAAATGAGCTGTTGAC | For amplification of Zeocin resistant gene |
| Rev_ZeocinR | TCAGTCCTGCTCCTCGGCCA |  |
| Fwd_F2 flanking region | GCCGAGGAGCAGGACTGATACTCGCGGTCACGTTTTTGTGCTTGTTTG | For amplifying F2 flanking region from *Methylomonas* sp. DH-1 genomic sequence |
| Rev_F2 flanking region | AAGCCTAAGCCTATGGCGATAAACA |  |
| Fwd_pET28_ *pmoD* | TGGACAGCAAATGGGTCGCGTATTCACACAGGAAACAGCTATGAAAAGACTATATAAATATGG | For amplifying *pmoD* from *Methylacidiphilum* sp. IT6 genomic sequence with overhang sequences for ligation into vector pET28a |
| Rev_pET28_ *PmoD* | GTCGACGGAGCTCGAATTCGTTATTCGTTATACTTATAACTATTCTGAACCATTC |  |
| Fwd_F1_Cyt P450_DH-1__EcoRI | CCACCTGACGTCTAGATCTGTGCCCTACGCCTTCGCGCTG | For amplifying flanks to knockout cytochrome P450, the PCR products were ligated into the EcoRI and SacI sites of digested pCM184 to construct pCM184-p450 |
| Rev_F1_Cyt P450_DH-1 _EcoRI | TCCATGGTACCAATTGTACAGCTGCCGATGTGCCGGGGAG |  |
| Fwd_F2_Cyt P450_DH1 _SacI | CGCGTGTTAACCGGTGAGCTCGGAATTCGCGAACTGGGCC |  |
| Rev_F2_Cyt P450_DH1 _SacI | GCTGGATCCTCTAGTGAGCTCACAGCTTCGGCGAGGTACGG |  |
| Fwd_qPCR_ p*moD* | AAGAACCCGGGAGACAGATA | For amplifying a segment of *pmoD* in RT-qPCR |
| Rev_qPCR_ *pmoD* | CCATTCTCCATAAAGCACCAATAAA |  |
| Fwd_qPCR_ref gene_*glgA* | TGGAAGGCAAACAGGCCAAT | For amplifying a segment of reference gene *glgA* in RT-qPCR |
| Rev_qPCR_ref gene_*glgA* | GTACTCTATGCTCTTGTCGC |  |
| Fwd_Cyt P450_DH1 | ATGCCTAACACCAGACCCGTAC | For amplifying cytochrome P450 coding gene in *Methylomonas* sp. DH-1 |
| Rev_Cyt P450_DH1 | TTAAGGTTTAGCCAACCGCT |  |

**Table S3.** Identification of proteins of the acetone assimilation gene cluster in *Methylacidiphilum* sp. IT6 and *Methylomonas* sp. DH-1 with the exclusion of particulate methane monooxygenase and novel PmoD protein

| **Protein Name** | **Gene** | **Locus Tag in IT6** | ***Methylacidiphilum* sp. IT6 (CP065957)** | **Locus Tag in DH-1** | ***Methylomona*s sp. DH-1  (CP014360)** |
| --- | --- | --- | --- | --- | --- |
| (Fe-S)-binding protein | *glcF* | IT6_09370 | WP_206826256.1 | AYM39_RS04895 | WP_064021222.1 |
| FAD-binding protein | *glcD* | IT6_09375 | WP_206826265.1 | AYM39_RS13600 | WP_064022087.1 |
| GMC family oxidoreductase large subunit | *gmcA* | IT6_09380 | WP_206826267.1 | AYM39_RS20165 | WP_064023073.1 |
| GMC family oxidoreductase small subunit | *gmcB* | IT6_09385 | WP_134439087.1 |  |  |
| VOC family protein (Glyoxalase) | *gloA* | IT6_09390 | WP_134439086.1 | AYM39_RS20250; AYM39_RS03110;AYM39_RS04650; AYM39_RS09045; AYM39_RS14435; AYM39_RS16860;AYM39_RS21550 | WP_064023086.1; WP_054761308.1; WP_064020699.1; WP_064021383.1; WP_054758599.1; WP_064022578.1; WP_064023289.1 |
| Hydroxyacylglutathione hydrolase | *gloB* | IT6_01870, IT6_08810, IT6_08820, IT6_09210 | WP_134440495.1; WP_206826112.1; WP_134439281.1; WP_134439120.1; | AYM39_RS06120 | WP_064020928.1 |
| phosphoenolpyruvate synthase | *ppsA* | IT6_09415 | WP_206826272.1 | AYM39_RS13170 | WP_064022016.1 |


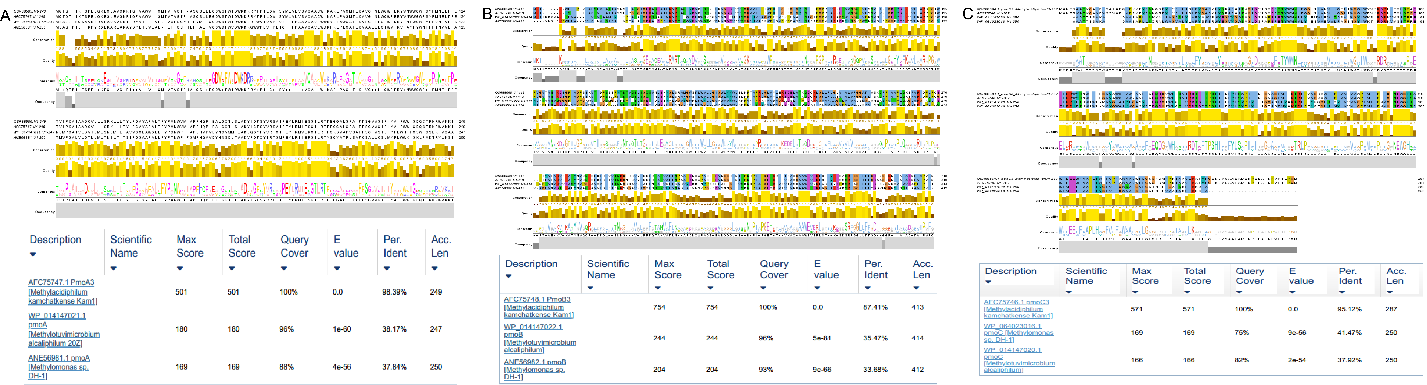


**Figure S1.** Multiple sequence alignment by Clustal Omega and BLASTP results of PmoA3 (A), PmoB3 (B) and PmoC3 (C) of *Methylacidophilum* sp. IT6 with corresponding proteins sequences of *Methylacidiphilum kamchatkense* Kam1, *Methylotuvimicrobium alcaliphilum* 20Z, and *Methylomonas* sp. DH-1.


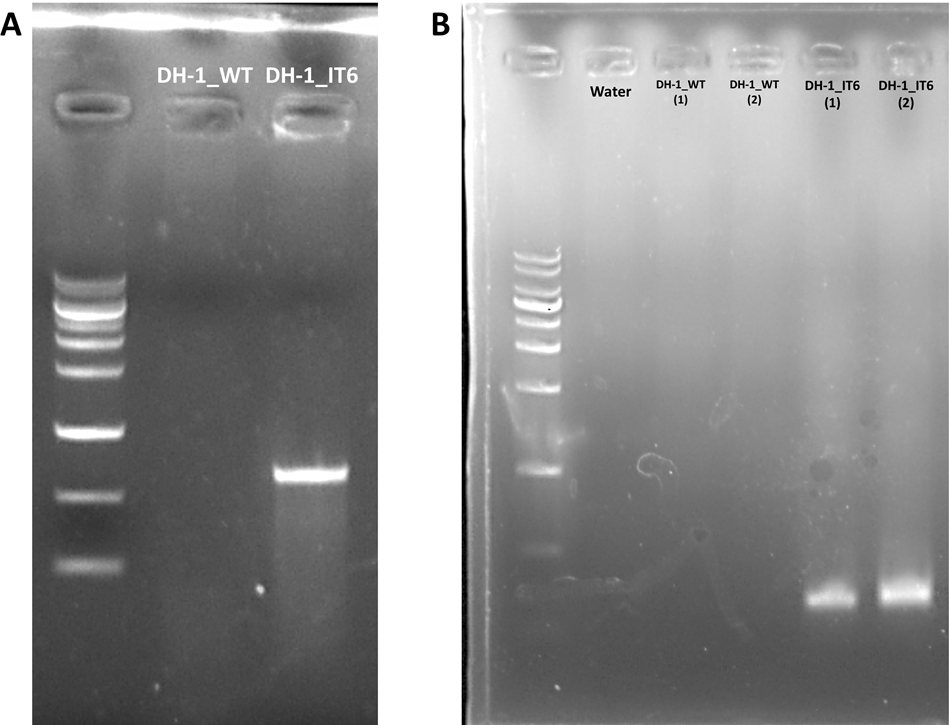


**Figure S2.** PCR results of *Methylomonas* sp. DH-1 wild-type (DH-1_WT) and recombinant (DH-1_IT6). A) Confirmation of the integration of *pmoD* of *Methylacidophilum* sp. IT6 into *Methylomonas* sp. DH-1 using the pair of primers to amplify the *pmoD*; B) Electrophoresis results of RT-PCR products to test the expression of the PmoD in *Methylomonas* sp. DH-1 recombinant.


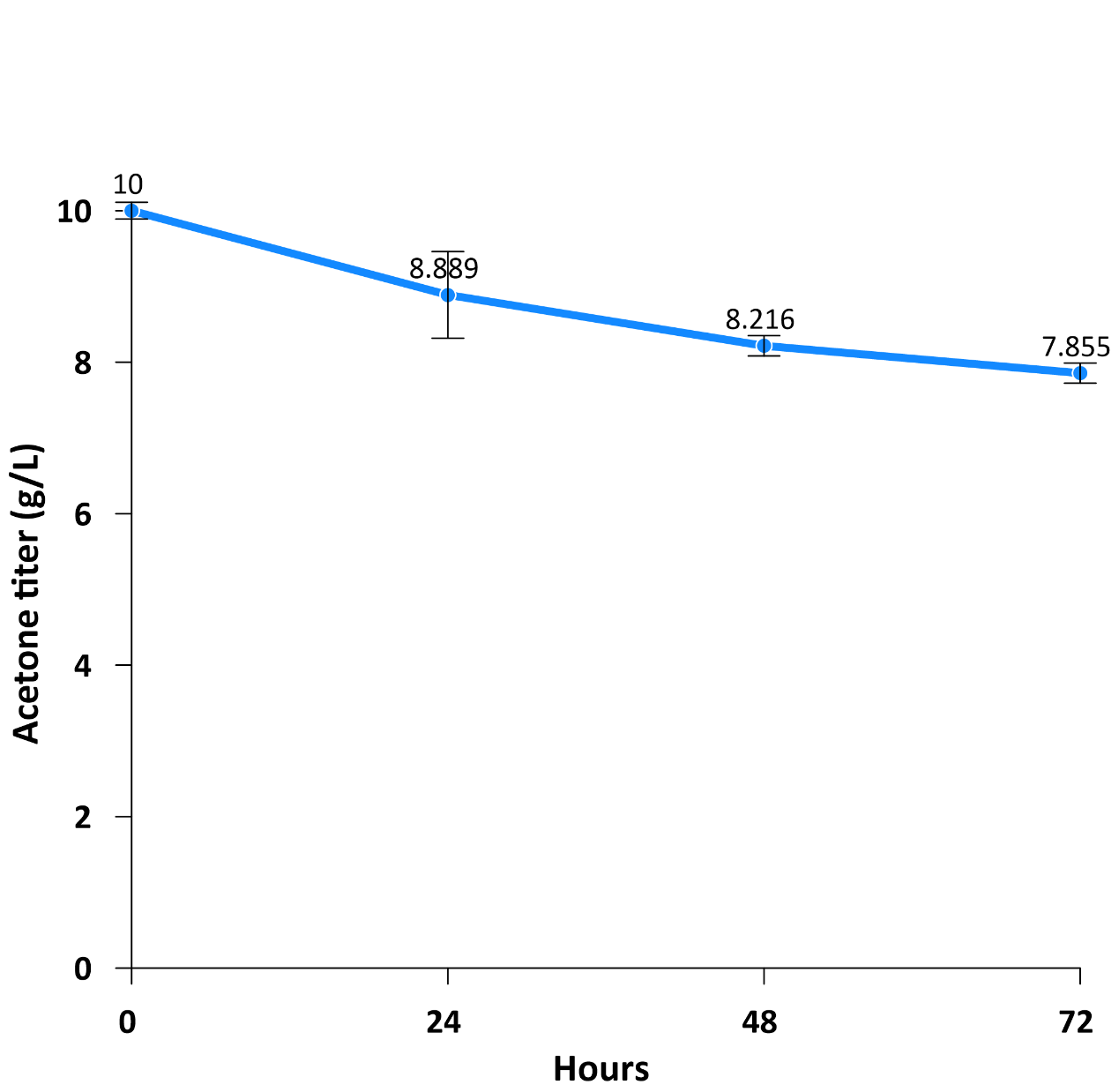


**Figure S3.** Acetone titers remained in 50 ml of NMS medium in the 500-ml baffled-flask sealed with a screw cap incubated at 30 °C and 250 rpm. The headspace was supplied with 30% (v/v) methane by a gas-tight syringe. The headspace was refreshed every day. Error bars represent the standard deviation. Three independent biological experiments were performed in triplicate and one representative experiment was chosen for figure production.


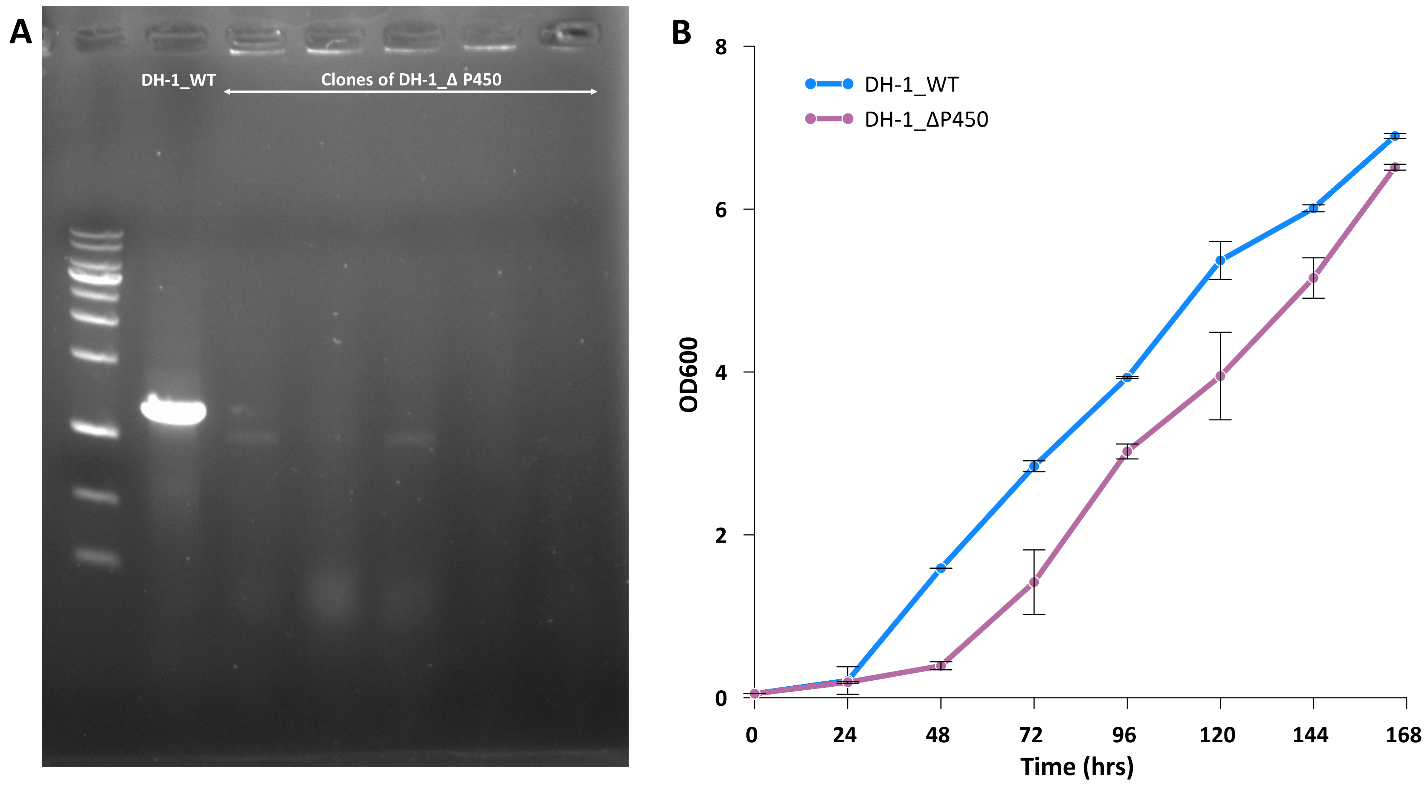


**Figure S4.** PCR results and growth rate of cytochrome P450-deleted *Methylomonas* sp. DH-1 (DH-1_ΔP450) A) Confirmation of the deletion of cytochrome P450 in *Methylomonas* sp. DH-1 using the pair of primers to amplify the cytochrome P450 coding sequence; B) Electrophoresis results of RT-PCR products to test the expression of the PmoD in *Methylomonas* sp. DH-1 recombinant. Growth rate (A) of *Methylomonas* sp. DH-1 wild-type (DH-1_WT) and recombinant (DH-1_ΔP450) strains cultured in 30% (v/v) methane. Error bars represent the standard deviation. Three independent biological experiments were performed in triplicate, and one representative experiment was chosen for figure production.

1. Awala SI, Gwak J-H, Kim Y-M, Kim S-J, Strazzulli A, Dunfield PF, et al. Verrucomicrobial methanotrophs grow on diverse C3 compounds and use a homolog of particulate methane monooxygenase to oxidize acetone. ISME J. 2021:1-12.
2. Hur DH, Na J-G, Lee EY. Highly efficient bioconversion of methane to methanol using a novel type I *Methylomonas* sp. DH-1 newly isolated from brewery waste sludge. J Chem Technol Biotechnol. 2017;92(2):311-8.
3. Marx CJ, Lidstrom ME. Broad-host-range cre-lox system for antibiotic marker recycling in gram-negative bacteria. Biotechniques. 2002;33(5):1062-7.
